# Are Partnerships With the Tobacco Industry and Food and Beverage Industry Possible? An Interview With Michael Eriksens

**Published:** 2009-03-15

**Authors:** Elizabeth Majestic

**Affiliations:** Centers for Disease Control and Prevention

Dr Michael Eriksen, former director of the Office on Smoking and Health at the Centers for Disease Control and Prevention (CDC) and director of the Institute of Public Health at Georgia State University, both in Atlanta, Georgia, was interviewed by Elizabeth Majestic with CDC's National Center for Chronic Disease Prevention and Health Promotion for *Preventing Chronic Disease*, CDC's online journal on public health policy, practice, and research (http://www.cdc.gov/pcd/). The Eriksen discussion focuses on whether the tobacco industry has forfeited its opportunity to participate in a traditional public-private partnership — the type that is sought by other industries — and if not, how it can partner successfully with the public health community. In the second half of his interview, Dr Eriksen considers the lessons learned from the tobacco control experience and how the public health community might work with the food and beverage industry to mitigate the obesity epidemic.

The interview was filmed in November 2008.

## Segment 1: Are public-private partnerships with the tobacco industry possible?

**Figure:**
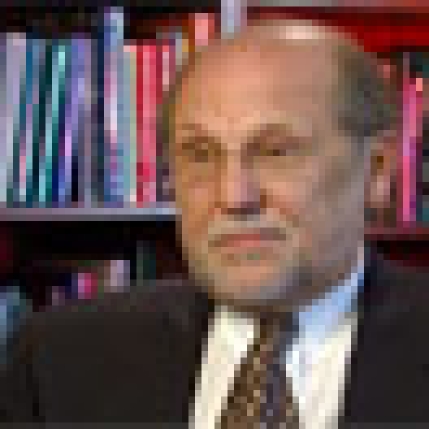


The tobacco industry has forfeited its opportunity to participate in traditional public-private partnerships, but does that mean that the public health community and the tobacco industry cannot work together to achieve a common objective of reducing the harm caused by tobacco use?

## Segment 2: What lessons, if any, should the public health community consider when forming partnerships with the business sector to mitigate the obesity epidemic?

**Figure:**
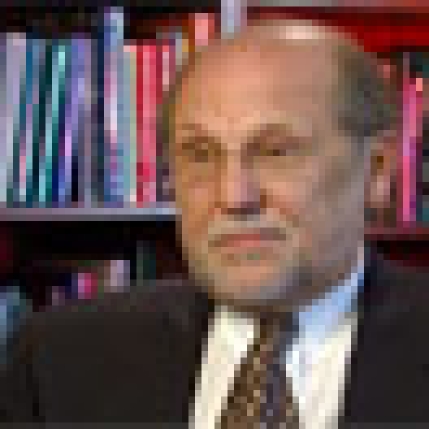


Partnerships with the private sector should not be about money, but rather, should be considered when they support the achievement of public health objectives. The public health community also needs to hold the food and beverage industry accountable for its actions. For example, food and beverage companies should focus their efforts on both physical activity and good nutrition and they should evaluate efforts based on public health outcomes.

## About Dr Eriksen

Michael Eriksen has been director of the Institute of Public Health at Georgia State University since 2002. He received his undergraduate and graduate training at the Johns Hopkins University and has had a long and distinguished career in public health. Dr Eriksen has been employed in academia (University of Pennsylvania, University of Texas, Georgia State University), the private sector (Pacific Telephone), state government (Maryland Department of Health and Mental Hygiene), federal government (Centers for Disease Control and Prevention), and international organizations (World Health Organization). He has published dozens of peer-reviewed articles on tobacco control, cancer prevention, and health promotion and is coauthor of The Tobacco Atlas with Judith Mackay. In 2004, the Georgia Cancer Coalition designated him as a Distinguished Cancer Scholar. Professor Eriksen teaches classes in the social and behavioral sciences, urban health, tobacco control, and global health. He is director of Georgia State University’s Partnership for Urban Health Research.

